# Exploring Levels of Interspecies Interaction: Expectations, Knowledge, and Empathy in Human–Dog Relationships

**DOI:** 10.3390/ani14172509

**Published:** 2024-08-29

**Authors:** Anna K. E. Schneider, Juliane Bräuer

**Affiliations:** 1 FAU Kompetenzzentrum für Interdisziplinäre Wissenschaftsreflexion (ZIWIS), Friedrich-Alexander-University Erlangen-Nuremberg, 91054 Erlangen, Germany; 2 DogStudies, Max Planck Institute of Geoanthropology, 07745 Jena, Germany; juliane.braeuer@uni-jena.de; 3 Department for General Psychology and Cognitive Neuroscience, Friedrich-Schiller-University of Jena, 07743 Jena, Germany

**Keywords:** companion animals, pet ownership, human–animal relations, qualitative analysis, interspecies communication

## Abstract

**Simple Summary:**

This qualitative study explores human–dog relationships with a focus on communication dynamics. Twelve human–dog dyads were analyzed using narrative interviews and cooperative tasks. The results suggest that human expectations shape interactions, transforming dog ownership into a family-like role. Effective communication relies on both verbal and non-verbal cues. Empathy is critical for deeper emotional connections. This study highlights that compatibility between humans and dogs has a significant impact on relationship satisfaction.

**Abstract:**

This exploratory study examines the complex dynamics of human–dog relationships and their impact on interspecies communication. Twelve human–dog dyads were studied using narrative interviews to explore how people perceive their relationships with their dogs. In addition, the dyads engaged in a cooperative task to observe interaction dynamics during everyday activities. This study shows that individual expectations frame interactions and that traditional notions of dog ownership are evolving into more family-like relationships. Effective communication relies on a nuanced mix of verbal and non-verbal cues, with empathy emerging as a fundamental element guiding these interactions. Our findings underline the profound influence of human expectations, knowledge and empathy on communication with dogs. They also highlight the critical role of compatibility between human and dog dyads, and emphasize that such compatibility is a key determinant of satisfaction in interspecies relationships. These findings contribute to a deeper understanding of how human factors modulate communication and satisfaction in human–animal interactions.

## 1. Introduction

Interspecies communication is an intriguing and burgeoning area of research. It explores the intricacies of how different species, particularly humans and non-human animals, exchange information and connect at various levels [1,2]. Historically, the idea of interspecies communication was met with skepticism regarding animals’ ability to communicate effectively. However, contemporary research now emphasizes the human side of this dynamic, focusing on how humans can adapt and improve their communication with non-human animals [3,4]. This shift, which recognizes that humans often hold preconceived notions about other animals, their states of consciousness, and their communicative capabilities, prompts a realization that “human beings are always in communication with other animals” [5]. This insight remains relevant even when individuals are not consciously aware of these interactions or find them challenging to fully comprehend. Furthermore, recent studies, such as the investigation by Bender et al. [6] into the determinants of guide dog–owner compatibility, underscore the importance of comprehending the factors that contribute to successful human–animal relationships and encourage a more inclusive examination that considers both sides of the communication dynamic between humans and non-human animals.

To ensure clarity, it is essential to define and distinguish key terms used in this study. Specifically, the terms interaction, communication, and relationship will be outlined. According to Abels, interaction is described as actions between at least two individuals that are related to each other [7]. This form of social action, as defined by Weber [8], is also referred to by Simmel [9] as interaction. Sociology distinguishes between normative and microsociological approaches to the emergence of interactions [7]. Since the 1980s, these terms have been extended to describe human–animal interactions [10,11,12]. In contrast, communication focuses on the transmission of information rather than just the coordination of behavior [13].

According to Rudy [14], effective interspecies communication requires recognizing the self–other boundary of non-humans within the interactions. This acknowledgment that, in many aspects, other animals will always be fundamentally ‘other’ to us, and their nature may never be entirely comprehended should foster respectful engagement, rather than be seen as an obstacle to meaningful connection. This highlights that for interspecies interactions to occur, humans and animals must first recognize each other as potential interaction partners. Initially, studies focused on expecting animals to demonstrate proficiency in human languages. This approach has since evolved to engaging with animals on their terms, recognizing and exploring the richness of non-human communication [15]. To tune into non-human communication effectively, we need to adopt a perspective that recognizes our interconnectedness with animals, which involves acknowledging their distinct ways of perceiving, interacting, and navigating their environments [16]. This process requires deep empathy, fostering a connection that transcends language [14]. In this article, we conceptualize empathy as an embodied and interactive process between humans and non-human beings, drawing on concepts such as embodied empathy, entangled empathy, and other nuanced understandings explored by scholars like Despret [17], Gruen [18], Shapiro [19], and Heddon [20]. For a comprehensive discussion of these perspectives, please refer to Schneider [21].

Throughout their shared life with humans, dogs have specialized in communication with their heterospecific social partners [22,23]. Most communicative skills do not appear to be learned, as they are evident from an early age, even in puppies [24,25,26]. For example, dogs continuously observe and respond to their human counterpart’s attention and adjust their behavior based on the human perspective [27,28,29]. Research shows that this extends to specific skills such as tracking the human gaze [30], as well as interpretating pointing gestures [31,32,33,34]. The context of a pointing gesture also plays a role for dogs, as a higher success rate has been demonstrated under cooperative conditions [35,36].

Beyond interpreting human behavior, dogs have also developed their own strategies for communicating information to their human social partners. These include vocalization [37] and physical manipulation [38,39,40,41], as well as the use of body movements or direction of gaze [42,43] or physically positioning at a location [44,45]. These communication tools are used both intentionally and with a referential function [46]. Beyond direct communication, dogs display a form of emotional contagion, mirroring or responding to the states of the humans with whom they share their lives [47,48], which reflects the most primitive form of empathy [49]. This contagion can also be seen in an analysis of physiological responses. For example, the quality of the relationship and the personalities of the caregivers, among other factors, influence the cortisone levels of the animal partners [50]. These emotional and physiological contagions emphasize the role of shared experiences and close bonds, bridging the communication gap between different species. Remarkably, these features are likely to contribute to establishing and maintaining an attachment bond, similar to attachment styles observed in child–parent interactions as in Ainsworth’s classification theory [51]. Research validated that dogs display attachment behavior toward their human, evidenced by exploration, play, and proximity seeking [52].

While dogs demonstrate an impressive ability to interpret human emotional cues, affective intensities, and shared experiences, the intricate nature of human–dog communication reveals a significant asymmetry [53]. In the context of interspecies communication, humans play a dual role in expressing love and attention to their dogs while also exercising authority over them to integrate them into human society [54], highlighting the pivotal role that humans occupy in shaping the dynamics of communication. To date, studies have explored the impact of human sensitivity to non-verbal communication, prior experience with dogs [55], and human familiarity on interactions with dogs [56]. Despite recognizing the dynamic, dual role of humans and highlighting their pivotal involvement in the process of interspecies communication, to our knowledge, the active contribution of humans to the formation and dynamics of connections with their dogs has not been clearly established. Therefore, the current study aims to gain a deeper understanding of the interaction dynamics between dogs and their owners while also exploring the key determinants that shape and influence these dynamics. The selection of the dog as the subject of this study is particularly ideal due to its unique capability for complex and sustained mutual interaction with humans [57,58].

## 2. Materials and Methods

The methodological framework chosen for the present study is qualitative social research. This approach is not merely concerned with examining an existing hypothesis but rather aims at a deeper exploration of social structures. Additionally, the focus is on investigating the subjective sensory worlds of both human and animal subjects [59]. Methodological triangulation (Denzin’s between method) was used to provide a comprehensive analysis of the research question, particularly in relation to cross-species participation. This involves systematic comparative analyses of observations and laboratory data for the objective description of the subjects’ actions, as well as interview analyses for the personal assessment of the interviewees and the recording of their subjective meaning [60]. The aim is to obtain different types of data and to combine them in order to complement the knowledge potential of the different approaches [60].

### 2.1. Participants

Twelve human–dog dyads participated in a laboratory study and interviews; all human participants were the primary caregivers and had lived with their dogs for a minimum of two years, with some having raised their dogs since puppyhood. The Department of Dog Studies at the Max Planck Institute maintains its own database containing information on dog owners interested in studies and details about their dogs (e.g., breed, age). This resource was used to recruit study participants. It should be noted that due to the qualitative nature of this study, although designed as a laboratory study, a smaller sample size was used. Given the exploratory nature of this study, purposive sampling and an “in-depth, interpretive exploration of the issues as they present themselves to people” [61] is preferred to quantitative representativeness. Both human and animal subjects were required to meet pre-defined selection criteria; so, a qualitative sampling plan (see Appendix A) according to Kluge and Kelle [62] was preferred to random selection. Human female participants aged 20 to 60 years from Jena (Germany) and the surrounding area and an equal number of untrained dogs of both sexes aged 3 to 10 years were included in this study. In this context, “untrained dogs” refers to dogs that have not undergone professional training, distinguishing them from working dogs or those with specialized training. These are typically pets without formal obedience or skill training. The final composition exhibited an average age of the dogs of about five and a half years and a wide variety of breeds, e.g., Eurasier, Golden Retriever, Bernese Mountain Dog, Tibetan Terrier, Pug, Bavarian Mountain Sweat Dog, and various mixed breeds.

### 2.2. Narrative Interviews

This study employed narrative interviews, which facilitate open and non-directive conversations and allow participants to share unconstrained stories [63] to delve deeply into the subjective perspectives of dog owners. Focusing on the owners’ personal interpretation of the relationship with their dogs, this approach aimed to uncover personal interpretations and discover previously unknown contexts. This allows us to gain valuable insights into the interplay between situational factors and personal experiences that influence action tendencies. The initial question asked participants to describe how they came to own their dog and their experiences since then. This broad prompt allows for a comprehensive account, touching on reasons for getting the dog and changes over time. The introductory prompt was supplemented with a guided inquiry section to ensure that all relevant aspects were addressed, thereby improving the comparability of the respondents’ answers. Example follow-up questions included inquiries about the dog’s relationship with household members, communication methods, and daily routines. The interviews were integrated into a broader laboratory study, with informal conversations during breaks to maintain a natural atmosphere.

We employed the laboratory task to observe human–dog dyads engaged in goal-oriented interactions in an indoor room setting. This behavioral study serves as a complementary approach to our interview-based research, functioning primarily as a triangulation method to enhance the robustness of our findings.

### 2.3. Laboratory Setting

To enhance this study’s comparability and feasibility, the laboratory setting and tasks were tested in a pilot study with dogs of various sizes, ensuring that all dogs could perform the tasks with appropriate guidance. The entire laboratory task was recorded on video.

**Figure 1:**
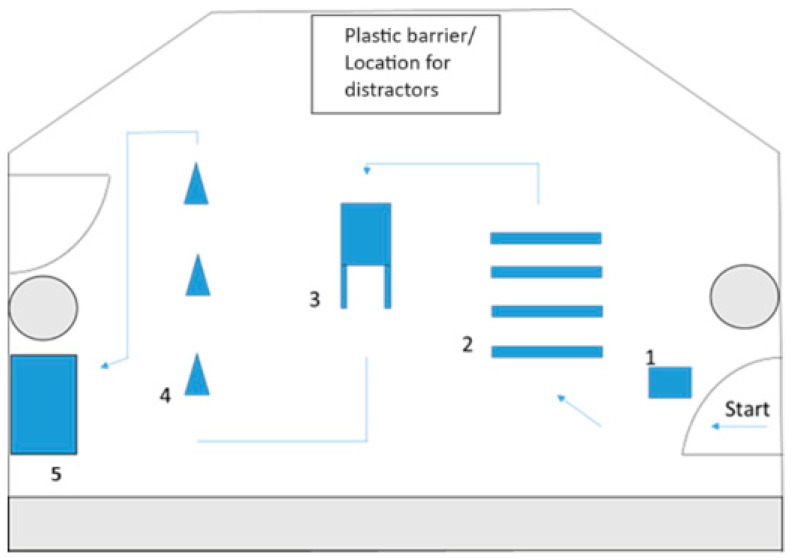
Layout of the laboratory room with course: The figure shows the design of the course in the room. The room is entered on the right side through the door and exited without external prompt after the participants feel they have completed the task. The blue symbols in the figure represent the various tasks as previously described: (1) marking a target, (2) negotiating a four-part cavaletti, (3) crawling between the owner’s legs, (4) slaloming around three pylons, and (5) lying down on a slightly elevated mat. The square box at the top represents the separate area behind the plastic barriers where the distractions are placed. The grey rectangle and circles indicate pillars and tables built into the room design, which are otherwise irrelevant to this study.

To allow for an extended observation period, this study incorporated five designated floor stations, each marked with numbers. The owner and their leashed dog entered the room, understanding that the entire exercise had to be performed off-leash. Tasks involved the dog sitting (see setup mark 1), negotiating a four-part cavaletti (2), crawling between the owner’s legs (3), slaloming around three pylons (4), and lying down on a slightly elevated mat (5). These tasks were always presented in the same order to ensure consistency (see Figure 1). Given that both humans and animals adapt their behavior to social environments and norms, influencing interspecies interactions, three conditions were included in the laboratory task to enhance the ecological validity of this study. A clear plastic boundary, either empty or containing a strange dog or person, was introduced to serve as a tactile barrier, allowing smell and sound permeability. The boundary was high enough to prevent any direct contact or jumping over it. All human–dog dyads completed the task with (i) no distraction (empty plastic boundary), (ii) human distraction (boundary containing strange person), and (iii) dog distraction (boundary containing strange dog). The distractors allowed us to assess potential disruptions in the interaction and to observe any deviations in communication within the dyadic interaction. Each owner–dog dyad participated in three sessions, with one session per condition.

The placement of the distractors behind the partition was chosen to prevent direct physical influence and safety-related conflicts. The same human and dog assistants were used consistently, with the human dressed identically in each repetition. The human assistant was instructed not to initiate interactions but to respond normally if approached by the participants. The selection of the dog assistant considered factors such as time availability, basic obedience, absence of aggression toward humans or other dogs, neutering, clear recognizability as a conspecific (e.g., size), and a basic interest in other dogs. This study was conducted on different days with two to five participants each day. The twelve dyads were divided into three groups to mitigate order effect bias. The repetition time was flexible, with a maximum limit of eight minutes to complete the entire course. Each run was followed by a minimum ten-minute break, utilized for interviews and to allow the dogs to relax.

### 2.4. Data Analysis

The transcriptions of the interviews were made with the aim of preserving the linguistic idiosyncrasies as accurately as possible, without approximating the written language. For the video material, there are two recordings from different sides of the room for each session. While video track 1 was primarily used for coding, the supplementary recordings from camera 2 were used to cross-check the material in case of uncertainties or missing recordings from camera 1, for example, due to an unfavorable angle. Strauss [64] describes coding as “a general term for conceptualizing data”; thus, coding involves asking questions about categories and their relationships and providing tentative answers (hypotheses) to them. Accordingly, concepts in the collected data were identified and abstracted into categories, after which hypotheses were generated about the existing emergences between the identified categories. On the basis of the coding paradigm developed, theory generation was carried out in an integrative manner in line with the additional data sets and their analysis.

For the analysis of the video material collected in the practical laboratory study, behavioral coding was performed using MAXQDA 12 software. A coding guide with different behaviors was developed for both parties (human and dog). The coding of the owners was based on the following five codes in two core areas: First, in the area of body language, (1) general physical orientation, i.e., orientation towards the dog, (2) bending or kneeling, and (3) use of hand signals; second, actual physical contact, with a distinction between (4) manipulative and (5) supportive touches. The dogs were coded comparatively using a coding scheme in the same core areas: In the body language category, (1) orientation towards the human and (2) static behavioral positions, such as sitting or lying down, were recorded.

Although we had initially hoped to code gaze direction from the video material, the recordings proved too imprecise for this purpose. Video-based time coding of vocalizations was also unreliable; so, manual observation protocols were used instead. These protocols analyzed changes in frequency, volume, type and expression of vocalizations. Subcategories included signals (e.g., “sit”), supportive speech (e.g., “it’s OK”), motivational speech (e.g., “good job!”) and corrections (e.g., “no”). Vocalization protocols were developed for human participants only as dogs produced few measurable sounds and were therefore excluded from this part of this study. Coding sheets were used as Supplementary Material in the final analysis.

To ensure reliability and minimize bias, several people were involved in the coding process. Codes and examples were first checked for clarity by an independent third party. The data collector initially coded the material and later recoded it to check intracoder reliability according to Mayring’s [65] standards. After confirming intracoder reliability, a third person coded the material to ensure intercoder reliability. The results were cross-checked for consistency and any uncertainties were resolved. The participants’ names and locations were anonymized using pseudonyms such as Mrs A and alternative names for dogs.

Following the Grounded Theory approach, we conducted a multistage coding process for the interview transcriptions, including coding sheets, observation protocols, and video coding results. All participating dyads were considered individual case studies and compared to the other cases. Adopting the approach by Strauss et al. [66], we categorized and summarized the concepts related to the phenomenon of interaction. Furthermore, a grouping of all concepts related to the phenomenon of interaction was developed. Subsequently, an insight into the further cross-case observations derived from the detailed analysis of individual case studies and overarching case comparisons is provided. Given the volume of data, not all categories will be exhaustively presented, with this study focusing on notable highlights through in-depth illustrations.

## 3. Findings and Discussion

With the main focus of this study being on interactions that occurred within pre-established human–dog relationships, three overarching themes emerged as key factors in shaping the nature and outcomes of human–dog interactions, each of which has significant relevance not only to the moments in this study but also to the periods leading up to and encompassing the participants’ relationships.

### 3.1. Expectations

The participants held varied expectations about their relationship with the dog, often shaped by childhood or previous experiences with animals, and dreams of living with their own dog. Even before direct interaction, the individuals were already ascribing roles to the dogs, contemplating whether they would be perceived as children, friends, or unspecified family members, emphasizing the belief that the dog must integrate into their lifestyle and form a unique emotional connection. The participants also pondered their roles as owners, grappling with questions of responsibility, time commitment, suitable living conditions, and considerations regarding specific dog breeds. As expected, the initial expectations are based on forming close relationships like parent–child role models.

In the case of Mrs. F. and Emma, the owner is a single parent, raising two daughters and refers to Emma as her “furry child”, which clearly underscores the special bond that the mother feels:

“It is my dog. So, I said from the beginning that I would get her because the children want her too, but she’s my dog”.

(Ms. F, lines 82–83)

Mrs. F, who engages in visitation work at a hospice center with her dog Emma, further illustrated this special bond as a connection that transcends routine or predictable patterns in their interactions:

“So, curiously, she always knows exactly when we’re going to the hospice. So, our walk round goes towards the hospice and turns off before. When we want to go to the hospice, [Emma] doesn’t turn, otherwise she basically turns. So, she knows we’re going, for whatever reason, I don’t know, because we don’t necessarily go at the same time”.

(Mrs. F, lines 215–219)

On the other hand, the case of Mrs. L and Lana demonstrates that the human–dog connection is not necessarily limited to the dyadic relationship of dog and owner. Additionally, a strong human partnership bond can be formed through the shared responsibility of caring for the dog, creating a distinct human “we” that coexists with the interspecies “we”, including the dog:

“And my other roommate, she is also a dog lover like me, um, we always have our room doors open, and then the dog can always decide, do I go to the one roommate or the other one […], she is the aunt, so to speak, yes”.

(Ms. L, line 102–105)

This dynamic extends beyond the primary owner, as the roommate is classified as an aunt, securing a defined inclusion in the human–animal relationship. Such immersion of individuals in the daily rhythms and interactions of life with the dog cultivates a profound understanding that goes beyond mere observation, establishing a foundation for meaningful and mutually enriching relationships, emphasizing the role of dogs as integral family members.

Of interest, it should be kept in mind that all the interviewees brought a desire for compatibility with the dog to the first contact because of the explicit decision to live with a dog. Therefore, the encounter with the dog as a counterpart begins unilaterally from the human side, before the other party could be aware of it. This establishes a framework (following [67])—one that remains open to modification—and serves as an organizing principle for subsequent interactions, even before the actual encounter takes place [68,69]. This also resonates with studies indicating that a majority of pet owners perceive their animals as close friends or kin, and recognize themselves as caretakers of animals, highlighting the pivotal role of human expectations in the complex process of interspecies communication [70].

### 3.2. Knowledge

Once the initial framework for interaction is set by the human side, the ongoing challenge of daily life and cohabitation unfolds. Interactions between human and their dog companions are primarily based on knowledge of humans about dog companions, establishing a bond structure. While prior knowledge of the species or a specific breed is beneficial, it also becomes evident that this knowledge alone is insufficient. Despite varied prior experiences with dogs or animals in general, all interviewees reported undergoing a learning process with their current dogs.

In a communicative context, varying levels of reflection on chosen communication, like spoken or body language, and strategies emerge, likely influenced by different knowledge levels. For instance, when asked how she communicates with her dog, Danilo’s elderly owner sees no need for alternative communication, stating unequivocally, “Well, through speech” (Ms. M., p. 141). However, most of the owners reported the use of purposeful, short commands, expressed through both verbal cues and body language, as essential for daily interactions and reinforced through training. The desire to incorporate body language often develops over time as people find that the dog understands them better:

“At first it was more linguistic when he was younger, but in the meantime, I’ve noticed that it works more with body language with him, and I’m also working on myself to communicate with him a bit more clearly”.

(Ms. A, lines 59–61)

This dynamic nature of communication within the human–dog relationship emphasizes the role of shared experiences and learning in fostering meaningful interspecies connections [71]. As human partners provide behavioral cues to their dogs, the dog partners develop communication strategies to express their needs. Commonly, participants state that dogs communicate a clear will or want message from the owners’ perspective, exhibiting direct, unambiguous, and persistent communication until their desired outcome is achieved. This process reflects engagement in a gradual process of developing a common language system to effectively exchange information.

A closer examination of individual cases shows that there is often an imbalance in the dog’s active participation in communication success. This imbalance can be attributed, in part, to dogs’ impressive ability to read and cooperate with humans during the domestication process, providing them with a clear advantage in understanding [72]. On the human side, factors such as a lack of knowledge about dog communication and the interference of expectations often contribute to misunderstandings, as highlighted by Haraway’s observations [16] on the challenges of taking the animal’s perspective. For instance, when a dog displays excitement, other dogs may assess it contextually, while humans might interpret it as positive or negative based on their individual needs. The dog often benevolently accepts or compensates for these communicative misunderstandings, emphasizing the importance of embodied knowledge in effective communication with dogs.

### 3.3. Empathy

Interspecies communication encompasses not only cognitive aspects but also emotional dimensions, contributing to a more comprehensive understanding and meaningful connection. This collaborative endeavor involves both parties, particularly when the dog’s behavior presents challenges for the owner, with underlying reasons not immediately evident. This scenario often triggers a reassessment of the human–dog interaction, prompting adjustments in communication methods. The emotional distress caused by the observable behavior, coupled with empathy for the dogs, initiates a quest for understanding the root cause and heightens sensitivity to the animal. For instance, Ms. L reported unsuccessful attempts to connect with the dog using her conventional methods, and eventually adjusted her approach, highlighting the significance of empathy in navigating successful communication:

“Because he is so difficult […], I didn’t really warm up to him at the beginning, it was […], at first it was just nice, he was just […] small and timid and he was looking for a lot of body contact with me, because he grew up, um, yes, probably without a mother, and then, however, in puberty, he became so exhausting that I became more and more frustrated, and somehow nothing really worked and what I normally do, how I normally train or discipline a dog, didn’t work at all with him. And that is, I had to relearn it, somehow completely different, so finally after a long time we found a dog trainer, who knows how these dogs tick, we have worked on it a lot, I am now warming up to him and I do really love him a lot now”.

(Mrs. L, Z. 157–169)

Similarly, Ms. E faces challenges with her second dog, Yilva, restricting her daily life due to problematic reactions to other dogs (Ms. E, lines 261–263). Despite unsuccessful training attempts, she has chosen to train Yilva for rescue work, which has transformed into a space for successful cooperation between them:

“But on the other hand, now, when I work with her, she is different, she is self-confident and asserts herself, she ignores me, she is supposed to, so, she is trained as a mantrailer, she is just completely different. She is really ambitious, strong-willed, she does her thing”.

(Ms. E, lines 51–53)

Despite the challenges, both exhibit a shared commitment to restructuring their daily lives for successful interactions with their dogs. Other instances within the collected material also support this observation. For example, Mrs. D’s husband works as a shepherd, and the family has “actually twelve sheepdogs” (Mrs. D, line 6). However, both are having trouble with Paula, who seemed uncontrollable from an early age on.

“Simply because when she was a puppy, I have to say, something did not, was wrong there, I do not know what it was. Cora, when the puppy was, she bit everything, she bit me, completely. So I had an open face, open hands, open legs, I no longer had long pants, she destroyed the entire furnishings of our apartment, but really from the table, to chairs, to stools and I then really did not know how to help myself”.

(Ms. D, line 50–57)

Despite experiencing physical and material damage affecting daily life, Mrs. D and her husband do not attribute blame. In fact, Mrs. D emphasizes that, irrespective of breed differences, it is about Paula as an individual that she states that “nothing worked”, which reflects reaching the limit of her knowledge. This highlights that when knowledge falls short, empathy becomes a valuable tool for comprehending the perspectives of others. Explicit understanding plays a crucial role in fostering openness to empathy and establishing the basic conditions for effective empathic responses. The case of Sam, the Pug, serves as an excursus, illustrating that, despite a high degree of empathy from the owner, misunderstandings can still occur when body language signs, especially in the presence of a second dog, are not recognized.
***Mrs. N and dog Sam****Sam’s case provides an interesting example of successful and unsuccessful communication between a dog and its owner during the laboratory task. Due to the nature of the task, most participants focus on the goal of completing the course, influencing communication styles geared toward guiding through, rather than listening. Mrs. N is a clear example of doing her best to find a way to communicate with Sam, adopting a low position, exercising patience, and emphasizing friendly body language. Sam comprehends the cues well, responding at varying speeds, creating the impression of a successful collaboration. Describing her interaction as being “on the same level as the dog” (Ms. N, lines 170, 177), she successfully guides Sam through the course in three conditions.**While Sam briefly hesitates upon entering the room with an unfamiliar human present, Sam displays behaviors that could be interpreted as signs of insecurity when faced with a second dog as a distractor. During a direct interaction with the distractor dog near the plexiglass, Sam’s stress seems to intensify, as evidenced by his retreat backwards, running to his owner, and seeking assistance by jumping up. Mrs. N briefly acknowledges Sam’s seeming distress before continuing with the exercise. Throughout the exercise, Sam exhibits uncertain body language and evasive actions, such as refusing to circle the pylon closest to the plexiglass. Ms. N acknowledges his refusals, but otherwise does not overtly react to his apparent discomfort.**Mrs. N, despite her strong focus, overlooks Sam’s emotional communication conveyed through body language. In the follow-up interview, she expresses surprise, expecting the second dog to be a greater distraction and anticipating a more assertive response from her dog. Ms. N’s expectation of self-confidence leads her to overlook Sam’s insecurity, highlighting the complexity of interspecies communication, where emotional signals may be misinterpreted, impacting the understanding of the dog’s needs.*

In the area of the empathic approach, two focal points can be distinguished. Some cases demonstrate a sincere effort to deal empathically with the dogs, but this willingness does not always yield the intended outcome. For example, humans often express positive affection through gestures like hugs, which can be misinterpreted by the dog. The dog’s body language might be misinterpreted, leading to inappropriate physical attention when the dog may prefer distance. Conversely, even if the need for closeness is accurately perceived, the dog might feel overwhelmed by predominantly human gestures like hugging, resulting in a misinterpretation of its need for social support. It should be noted here that misunderstandings in interspecies communication are not exclusively human-driven; animals, in this case, dogs, also communicate social support in a species-specific way, which may not always align with human preferences. Humans, for example, often find it challenging to interpret dogs’ emotional cues and frequently underestimate the impact of their heightened senses of smell and hearing that shape the dog’s sensory experience [73]. In the context of human–dog communication, these heightened sensory experiences might lead to dogs communicating in specific ways that humans may not fully understand, contributing to the communication challenges faced by human–dog dyads within this study.

## 4. General Discussion

In the current study, we focused on identifying different ways of initiating and maintaining interaction between dogs and their owners, as well as different communication styles. As the interview analysis shows, the human–dog relationship is primarily shaped by the expectations, knowledge, and empathy of the owners.

Our first observation was that expectation sets the framework for human–dog interaction. The acceptance of a dog into the household is not merely a practical arrangement but is an individually justified decision of the human party to extend their daily life with the company of an animal. The shift in recognition of animals as mere ‘pets’ to ‘companion animals’ and their human counterparts not as ‘owners’ but as ‘caretakers’ underscores the evolving family-like expectations humans have for their dogs [74]. The term caretaker and dogs’ reliance on their humans for basic needs align with the observed special, parent–child-like attachments that develop between humans and their dogs. From an alternative perspective, dogs might be viewed as reflections of human identities or social status [75], contributing to the establishment of expectations regarding appropriate human–animal relationships [76]. Treating dogs as family members underscores the recognition of their subjectivity and otherness. In this context, the recognition of subjectivity might be a cornerstone for enhancing human–dog relationships.

Secondly, it is not surprising that we observed the importance of prior knowledge and understanding of their species or breed for effective communication with dogs. However, it becomes evident that this knowledge alone might be insufficient in establishing meaningful connections. As Haraway [16] emphasizes, interspecies communication is not easy and relies on a combination of embodied gestures, learned commands, and environmental circumstances. This is analogous to navigating conversations with individuals who speak different languages in which non-verbal cues serve as bridges for mutual understanding [15]. Similarly, human–dog interaction demands a multifaceted approach. It is not merely about mastering a set of commands but attuning ourselves to the lived experiences of dog companions. This attunement is a dynamic process that evolves over time, requiring sustained effort and a willingness to engage in the shared embodied “language” that develops through years of interaction [77]. Through such shared learning and embodied experiences, a reciprocal exchange of knowledge is cultivated, nurturing a pathway for mutual communication.

In exploring interspecies communication, it becomes evident that the forms of embodied communication extend beyond our human imagination. Dogs, for instance, adeptly utilize sounds, gestures, and scents to convey messages, revealing a diverse range of communication modalities [78]. However, our human sensory capabilities are limited in perceiving the intricacies of their sensory worlds. This limitation becomes a crucial factor in potential miscommunications with non-human species, stemming from challenges in understanding and resonating with their unique sensory experiences. Taking domesticated dogs as an example, their olfactory features are specialized to discern specific human emotions, such as anxiety, showing the nuanced ways in which the human–dog relationship unfolds. While we cannot fully know the sensory experiences of other animals, our efforts to attend to such sensory modalities contribute to forming a shared version of language.

Given the role of shared experiences and intricate connectivity with the human–dog relationship as mentioned above, it is not surprising that the final observation was the crucial role of empathy in guiding the human–dog relationship. By adopting an empathetic approach, we go beyond mere observation; we aim to understand and resonate with the emotional and experiential dimensions of animal companions [79]. Notably, it becomes essential to not only understand but also respect the inherent differences and ‘otherness’ of animals [14]. This idea resonates with the concept of affection attunement, indicating the connection formed through shared emotional experiences, cultivating a heightened attentiveness towards other animals [79]. Since we exist in close relation to our dogs, we become entangled with them, and the nature of our interactions with dogs becomes a profound reflection of this interconnectedness [80]. The essence of entangled empathy lies in the mutual shaping of identities through their interactions. This notion aligns with a historical perspective that challenges the idea of a distinct and individualized self, emphasizing a blurred boundary between human and animal realms [81]. Haraway’s perspective suggests that both humans and animals have evolved through their engagements with each other and the practices that facilitate specific connections to emerge and develop. The interconnectedness of our identities unfolds through shared experiences, challenging notions of distinct individualized selves, and emphasizing the co-constitutive relationships that shape both humans and dogs.

As we explore the intricate terrain of human–dog interactions, the asymmetry inherent in these processes becomes quite clear. Humans emerge as central architects, wielding significant influence over the nature of experiences shared with dogs. Thus, we are compelled to delve deeper into the nuanced interplay of human attitudes, skills, and knowledge, recognizing their profound modulating effects on our behavior towards animals. This recognition is not merely academic discourse; it holds practical implications for fostering positive interactions, promoting animal welfare, and enhancing the overall well-being of both humans and their four-legged companions [82]. By understanding the modulating effects of human factors, we pave the way for a more harmonious and mutually enriching coexistence between species, where the welfare of each being is intertwined with the other. Simply put, this emphasizes the significance of human–dog relationships for fulfilling partnerships with a shared journey towards well-being.

The current study makes valuable contributions to the dynamics of interaction between humans and dogs; however, it is essential to acknowledge the inherent limitations of this study. Primarily, this study’s findings are drawn from twelve participants, which may limit the generalizability of the findings to a broader population of dog owners. Secondly, while this laboratory study provided controlled conditions for goal-oriented interactions, it might not fully capture the richness and complexity of real-world, day-to-day interactions between humans and their dogs. These limitations and the exploratory nature of this study necessitate follow-up studies for a deeper analysis of the variability in the theoretical approach.

## 5. Conclusions

The present study aims to explore the multifaceted dynamics of human–dog relationships, highlighting key elements that shape and define these interactions. Expectations, knowledge, and empathy emerge as pivotal forces steering the course of human–dog communication. Evolving family-like expectations foster unique parent–child-like attachments. Prior knowledge about dogs provides the basis for effective communication, yet our findings emphasize that true connection requires a multifaceted approach to both verbal and non-verbal cues, contributing to the formation of a shared communicative space. Finally, empathy emerges as a building block, guiding human–dog relationships beyond observation to a deeper understanding of the emotional and experiential dimensions.

The pilot nature of this study suggests the need for follow-up studies to explore the variability of the theoretical approach in greater depth and to expand on related themes. Comprehensive insights will require both highly specialized individual studies and broad theoretical integration. In addition, the influence of socio-cultural factors on human interaction strategies and environmental factors on animal behavioral preferences should be considered. The specific context of interactions and its impact on behavior is also crucial for understanding asymmetric or symmetric interspecies relationships.

## Data Availability

The data presented in this study are available on request from the corresponding author due to privacy and ethical reasons.

## Supplementary Materials

The following supporting information can be downloaded at: https://www.mdpi.com/article/10.3390/ani14172509/s1, Coding Sheet–Labratory Study–Interactive Parcours.
