# Supplementary material for: Exploring Levels of Interspecies Interaction: Expectations, Knowledge, and Empathy in Human–Dog Relationships
Source: Animals (Basel). 2024 Aug 29;14(17):2509. doi: 10.3390/ani14172509 (PMC11394575; doi:10.3390/ani14172509)
Supplement: Supplementary file 1 [file animals-14-02509-s001.zip › animals-3114986-supplementary.pdf]

Coding Sheet – Labratory Study – Interactive Parcours

**Category**	**Definition**	**Example**	**Rule**
Interruptions			
IH:<br>Interruptions human	Human interrupts the interaction and increases distance to the dog by walking away from him.	Human walking towards other object, door or generally increasing distance.	Coding starts with turning/moving away from human until he turn back to the dog/faces the dog again.
ID:<br>Interruptions dog	Dog interrupts the interactions and increases the distance to the human by walking away from him.	D walking towards other object, door or generally increasing distance.	Coding starts with turning/moving away from human until he turns back to the human/faces the human again.
Body language			
BH1:<br>Hand movements	Human moves hand to point, lure or give signals.	Human points towards chair etc.	Coding includes the whole movement.
BH2:<br>*Facial expressions (human)*<br>➔ *Excluded due to difficulty coding the video material*	*Human uses a noticable facial expression to communicate.*	*Laughing, looking very stern at dog for a longer time period etc.*	*Duration of the facial expression*
BD1: Facial expressions (dog)<br>➔ *Excluded due to difficulty coding the video material*	*Dog uses facial expression to communicate.*	*Flash teeth, swelling of muzzle etc.*	*Duration of the facial expression.*
BH3: Facing dog	Upper body leans towards dog.		Coding may include facing the dog partly while walking.
BD2: Facing human	Upper body faces human or hand signal.		Coding may include only facing the human with the head.
BH4: Crouching down (human)	Crouching down, bowing down to level of the dog (upper body tilted at least 90 degrees) when in interaction with dog	Includes kneeling etc. Excludes time spent taking off leash/putting on leash	From starting to crouch down to getting back up.
BD3: Sitting, Lying down	Dog rests on the ground.	Includes sitting down, laying down etc.	Duration of the position being held.
Touch			
TH1: Supporting touch (human)	Supporting touch.	Petting, calming touches, help with balancing etc.	Coding starts and ends with physical contact.
TH2: Manipluating touch (human)	Manipulating the dog’s body.	Pushing the body in a certain direction, moving paws or	Coding starts and ends with physical contact.

		body parts, moving the dog's body around etc. Also using the leash or harness to manipulate the body.	
TD1: Initiating physical contact (dog)	Initiating physical contact with human.	Pushing with nose, leaning body on leg, pawing, nuzzling etc.	Coding starts and ends with physical contact.
Vocal Communication			
VH1a: Active: Signals<br>VH1b: Reactive Signals	Giving the dog vocal signals/trained commands.	Signals like "sit", "stay" etc.	Coding only the vocalization.
VH2a: Active: Supportive Talk<br>VH2b: Reactive: Supportive Talk	Talking to the dog to calm or relax it.	Soft whispers, confirmations, "everything is fine" etc.	Coding only the vocalization.
VH3a: Active: Encouraging Talk<br>VH3b: Reactive: Encouraging Talk	Talking to the dog encouragingly.	High voice, enthusiastic talking, "that's great" etc.	Coding only the vocalization.
VD1a: Active: Barking<br>VD1b: Reactive: Barking	Barking	Barking	Coding only the vocalization.
VD2a: Active: Whining<br>VD2b:Reactive: Whining	Whining noises	Whining noises	Coding only the vocalization.
VD3a: Active: Snorting, Heavy breathing<br>VD3b: Reactive: Snorting, Heavy breathing	Snorting, heavy breathing	Snorting, heavy breathing	Coding only the vocalization
VD4: Reactive: Follows the commands	Dog follows the signals/commands.	Dog is told to "sit" and sits down.	Coding the process of fulfilling the command.
